# External Toxidermias

**Published:** 1919-07-12

**Authors:** 


### THE NEW DERMATOLOGY.

### III. External Toxidermias.

A CONSIDERABLE proportion of those who attend the dermatological clinics of our hospitals are suffering from dermatitis produced by irritant substances, to which they have been exposed either in certain industrial occupations, or in the performance of their ordinary domestic work. One of the commonest examples of such "occupation" or "application" dermatitis is that seen among washerwomen, due to the continued action of water, soap, washing soda, &c., on the skin of their hands and forearms; the alkali, in particular, dissolves the protective fatty substances from the skin, the horny layer becomes dry and cracks, with the inevitable result that micro-organisms, particularly staphylococci, are enabled to multiply in the damaged areas, and a dermatitis of varying intensity ensues.

But even an example, so apparently simple as this, presents points of great interest, for, if a case be investigated, it will usually be found that the patient has "washed" for years without any untoward result, save for an unnatural dryness and roughness of her hands, but that, when once she has experienced an acute attack of dermatitis, her resistance remains permanently lowered, and, if she must continue her work, her dermatitis becomes more or less a chronic condition, with periodical acute exacerbations.

Another point that has to be considered in these cases is the rôle played by the bacteria, which are enabled to take an active growth when the skin has already been damaged by an irritant. For not only does the person affected develop hypersusceptibility to the irritant, whatever it be, but also to the organism responsible for the secondary infection, usually a white or yellow staphylococcus, sometimes apparently a streptococcus. The following case illustrates this: A colleague of the writer developed a severe dermatitis of his hands and forearms, due to the constant use of carbon bisulphide and formalin in pathological work; as a result of this, secondary infection with a staphylococcus aureus took place. He was treated with an autogenous vaccine, from which he obtained great benefit, the secondary pyogenic infection subsiding but a mild form of the original dermatitis remained, which yielded to the use of a paste containing 4 per cent. salicylic acid. No doubt exposure to carbon bisulphide and formalin would again provoke an acute outbreak of the dermatitis, but it is hardly justifiable to recommend the experiment. In this case there were evidently two factors, viz., the original irritant, and the secondary bacterial infection; a vaccine was successful in overcoming the latter, but had no influence on the original cutaneous reaction to the carbon bisulphide-formalin.

The "internal" factor, too, even in cases of external toxidermia, is of great importance, for it will usually be found that the acute outbreaks of dermatitis appear when the patient is in a poor state of health. Another case may be briefly described to illustrate this point: A few months ago the writer was consulted by a woman, who had a severe eczematous eruption of the pompholyx type, involving her hands and spreading up her arms. Secondary infection of the vesicles had occurred, and a staphylococcus aureus was isolated in pure culture; cultivations from newly-formed vesicles, however, gave no growth at all. Apart from the eruption on her hands and arms, there were symmetrical areas of acute dermatitis over both breasts, due to the application of belladonna plasters with a view to arresting the flow of milk. The exciting cause of the dermatitis on the hands and arms was probably washing soda. The patient was evidently in a state of profound toxæmia; she was pigmented, she perspired excessively, and her temperature was raised two or three degrees. Examination showed severe oral sepsis, visceroptosis with lax abdominal walls, hypochlorhydria, and very marked indicanuria. Treatment was directed towards counteracting the toxæmia; several septic teeth were extracted and an antiseptic mouthwash prescribed, full doses of hydrochloric acid after meals with morning saline purgatives were given, and the patient was supplied with an abdominal belt in order to relieve her visceroptosis. Rapid improvement followed, and at the present time she has lost her toxic appearance, she has gained very considerably in weight, and, although she has resumed her household duties, she has had no recurrence of her eruption. This case illustrates very well the fact that a person in a state of acute toxæmia shows a heightened susceptibility.

In this connection it is hardly irrelevant to insist once more on the susceptibility also shown by persons of the seborrhœic diathesis (or, if objection be taken to the word "diathesis," we may say persons who show a tendency to seborrhœic eczema, acne, &c.) to such external irritants. This fact is of importance on account of the readiness with which these persons develop secondary dermatitis when substances such as sulphur, pyrogallic acid, mercury, and chrysarobin are applied to their skin. Among our troops on active service it was found that the men in whom infection with scabies was followed by severe dermatitis were usually of the seborrhœic type, and, as they were also extremely susceptible to the irritant effect of sulphur, treatment was rendered very difficult.

As it would be unprofitable to attempt to give an account of all the different types of irritants which may provoke an "application" dermatitis—ranging as they do from poison plants to hair-dyes—only two will be dealt with, namely, chrysarobin and dichlorethyl sulphide ("mustard-gas" or "yperite"). These two substances resemble each other very closely as regards their action on the skin and mucous membranes, and in the next article their properties and mode of action will be considered in detail. (*To be continued.*)

☛ **Previous articles appeared on May 17 and June 7, p. 239.**

